# An autopsy-confirmed case of progressive supranuclear palsy with predominant postural instability

**DOI:** 10.1186/s40478-016-0391-7

**Published:** 2016-11-14

**Authors:** Carolin Kurz, Georg Ebersbach, Gesine Respondek, Armin Giese, Thomas Arzberger, Günter Ulrich Höglinger

**Affiliations:** 1 Psychiatric Clinic, Psychiatrische Klinik, Ludwigs-Maximilians-Universität München, Nußbaumstr. 7, 80336 Munich, Germany; 2 Hospital for Movement Disorders and Parkinson’s Disease, Straße nach Fichtenwalde 16, 14547 Beelitz-Heilstätten, Germany; 3 Department of Neurology, Technische Universität München, Ismaningerstr. 22, 81675 Munich, Germany; 4 German Center for Neurodegenerative Diseases e.V. (DZNE) Munich, Feodor-Lynen Str. 17, 81377 Munich, Germany; 5 Center for Neuropathology and Prion Research, Ludwig-Maximilians-University Munich, Feodor-Lynen-Str. 22, 81377 Munich, Germany

**Keywords:** Progressive supranuclear palsy, Atypical clinical phenotype, Postural instability, Neuropathology, Magnetic resonance tomography

## Abstract

Postural instability and supranuclear gaze palsy represent the key symptoms of Richardson’s syndrome, the most frequent clinical manifestation of progressive supranuclear palsy (PSP). However, a proportion of PSP patients never develops ocular motor symptoms, which prevents clinicians from establishing the diagnosis during lifetime according to current diagnostic criteria. We present one instructive autopsy-confirmed PSP case with prospective video-documented clinical course, showing striking temporal divergence of initially present postural instability and delayed development of ocular motor dysfunction. Brain imaging and autopsy findings were typical of PSP, but the temporal sequence of symptoms was unusual with isolated postural instability predominating the clinical course for many years and slowing of vertical saccades/supranuclear gaze palsy evolving not until the 9^th^/11^th^ year after disease onset. Although other differential diagnoses were unlikely, this patient did not pass the threshold for possible or probable diagnosis of PSP according to current diagnostic criteria until very late in the disease course. This first well documented, autopsy confirmed case of PSP with predominant postural instability further expands the clinical spectrum of PSP and points out the need of new clinical diagnostic criteria with sufficient sensitivity and specificity for an early and reliable diagnosis.

**Electronic supplementary material:**

The online version of this article (doi:10.1186/s40478-016-0391-7) contains supplementary material, which is available to authorized users.

## Background

Progressive supranuclear palsy (PSP) is a disease entity defined neuropathologically by aggregates of the microtubule associated protein tau in astrocytes (tufted astrocytes), neurons (neurofibrillary tangles) and oligodendrocytes (coiled bodies) in typical anatomical distribution [1, 2]. Postural instability (PI) with falls, and slow vertical saccades followed by supranuclear gaze palsy (SNGP) represent the key symptoms of the typical clinical manifestation of PSP - termed Richardson’s syndrome (PSP-RS) [3, 4]. The NINDS-SPSP criteria for the clinical diagnosis of PSP require a combination of PI during the first year and slowing of saccades or SNGP [5]. Proportions of 9–59 % of autopsy confirmed PSP patients have been reported to never develop SNGP throughout the disease course [3, 6–9]. The causes for these variable and high numbers remain unclear. Atypical clinical manifestations of PSP have been described, e.g. PSP with initially predominant Parkinsonism (PSP-P), frontotemporal dysfunction (PSP-FTD), or akinesia with gait freezing (PSP-PAGF), in which SNGP may indeed evolve later in the disease course [10]. A recent retrospective study of *N* = 100 autopsy confirmed cases has also suggested that as many as 18 % of PSP patients may have an abortive development of PSP-RS, with PI predominating the early clinical course and SNGP only developing with major delay, coining the term PSP-PI. The existence of PSP-PI cases has been questioned, however, since an apparent lack of SNGP in retrospective series might result from insufficient examination or documentation of the actual clinical symptoms. Here, we report the first prospectively video-documented patient with progressive PI predominating the clinical course for many years and very late onset of SNGP, having confirmed PSP at autopsy, supporting the concept of PSP-PI. This patient did not pass the threshold for possible or probable PSP according to current diagnostic criteria until very late in the disease course [11].

## Case presentation

**Fig. 1:**
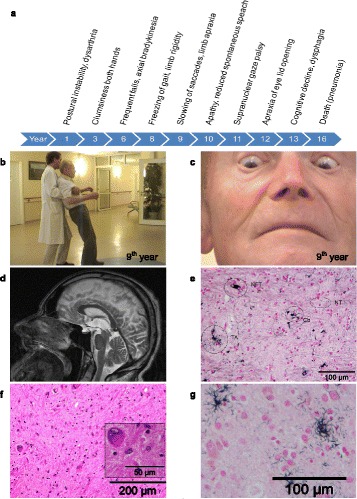
Case presentation. **a** Timeline of the evolution of clinical symptoms. **b** and **c** Demonstration of absent supranuclear gaze palsy (SNGP) despite clear postural instability (PI) in the 9^th^ year after symptom onset. **d** MRI showing predominant midbrain atrophy in the 13^th^ year after symptom onset. **e** Representative histopathological section (pallidum) showing the typical histopathological hallmarks of PSP), i.e. tufted astrocytes (TA), coiled bodies (CB), neurofibrillary tangles (NFT) and neuropil threads (NT) (Gallyas silver stain). **f** Histopathological section (globus pallidus) with loss of neurons, intracytoplasmic inclusions and gliosis (Hematoxilin-Eosin staining). **g** Tufted astrocytes in striatum (globus pallidus) as the neuropathological hallmark of PSP (Gallyas silver stain)


The temporal evolution of symptoms is shown in Fig. 1a and in the supplementary video (Additional file 1). At 67 years of age (in 1997), the initial symptoms occurred: PI, pain of the left shoulder, hypokinesia of the left arm, hypomimia and dysarthria. In the early disease course the gait was minimally affected, it was slightly broad based and slowing of turning was noted, the patient did not require walking aids. The first falls occurred backwards in 2000, the patient had no gait ignition failure. At first, Parkinson’s disease (PD) was suspected. After 5 years, atypical Parkinsonism was considered, since the patient showed frequent falls and was non-responsive to levodopa. The patient showed slight, but not pronounced micrographia and hypohonia in the beginning of the disease. Between year 5 and 10 after disease onset, the patient developed mild limb rigidity, symmetric bradykinesia, apraxia of both hands, freezing of gait and apathy. Slowing of vertical saccades despite full range of voluntary ocular movements in all directions was first observed in the 9^th^ year of his illness, vertical SNGP was only observed in the 11^th^ year (Fig. 1b and c, Additional file 1). Apart from bilateral limb apraxia in the late course, the patient did not show other signs of cortical sensory loss. The patient’s condition worsened due to dysphagia and cognitive decline in the 13^th^ year of the disease. The patient died of aspiration pneumonia after an exceptionally long disease duration of 15 years at the age of 82.

Additional file 1: Video-documented clinical course of an autopsy-confirmed PSP patient with predominant postural instability (PSP-PI) and very late onset of ocular motor dysfunction. In 1997, the initial symptoms occurred at the age of 67. The patient was filmed three times. *In 2005 (9* ^*th*^ *year after onset)* postural instability and freezing of gait was observed. In *2006 (10* ^*th*^ *year after onset)* gait became further unstable despite full range of voluntary ocular movements. In *2009 (13* ^*th*^ *year after onset)* the patient showed supranuclear gaze palsy, apraxia of eye-lid opening and cognitive decline. (MP4 12617 kb)


### Brain imaging

Single photon emission computed tomography (SPECT) with ^123^J-FP-CIT in the 8^th^ year revealed bilaterally reduced striatal dopamine transporter density. Magnetic resonance imaging (MRI) showed predominant atrophy of the midbrain tegmentum (anteroposterior midbrain diameter of 11.9 mm after 12 years and 11.6 mm after 14 years) with enlarged ventricular spaces, moderate prefrontal atrophy and discrete vascular lesions (Fig. 1d). The midbrain-to-pons-ratio was 0.49 [12].

### Autopsy findings

Although the temporal evolution of symptoms was unusual, brain autopsy findings revealed typical neuropathological features of PSP: Macroscopically, slight frontotemporal and severe midbrain atrophy was observed, furthermore dilated lateral ventricles. While substantia nigra, dentate nucleus (cerebellum), locus coeruleus and medulla oblongata exhibited pronounced neurodegenerative changes (neuronal loss and gliosis) on haematoxylin-eosin-histology, globus pallidus showed only discrete neurodegenerative changes. Tau immunostaining demonstrated widespread tau deposits in tufted astrocytes (TA), coiled bodies (CB), neurofibrillary tangles (NFT) and neuropil threads (NT) in frontal cortex, striatum, insula, basal ganglia, amygdala, hippocampus, midbrain and pons. Thus, the neuropathologic criteria for PSP were clearly fulfilled (Fig. 1e) [2]. The tau distribution fits into score 4-5 according to the scale presented by Williams with more severe involvement of the basal ganglia and dentate nucleus with involvement of the frontal and parietal lobes [13]. Tufted astrocytes were mainly found in the caudate nucleus (severe) and frontal cortex (moderate) and transentorhinal cortex (moderate). The following concomitant neurodegenerative pathologies were observed: widespread beta amyloid deposits (stage 5) [14], cerebral amyloid angiopathy (1 type 2) [15], Alzheimer’s disease (Braak and Braak stage 3) [16] and argyrophilic grain disease (stage 3) [17]. The “ABC” score for AD neuropathologic changes in this case was A3 B2 C2 [18]. TDP43 positive deposits were not detected in the following brain regions: frontal cortex, striatum, hippocampus and amygdala, caudal medulla and cerebellum.

## Conclusions

While SNGP was initially described, and is still considered as the most specific clinical hallmark of PSP, coining the disease’s name, we present to our knowledge the first prospectively video-documented and autopsy-confirmed PSP patient with an unexpected long latency of 11 years between the clinical disease onset with initially predominant PI and late development of SNGP. This patient provides clear evidence for the concept that a subgroup of definite PSP does indeed present with a clinical predominance type of PSP-PI [5]. We carefully considered the possibility of this case representing a variant of pure akinesia with gait freezing (PAGF), which is considered to be one of the phenotypic presentations of pallido-nigro-luysial atrophy (PNLA), a rare variant of PSP [19, 20]. The long disease duration of 15 years and the late onset of SNGP fit the prior descriptions of PNLA/PSP-PAGF [20]. However, early onset of falls, late onset of freezing of gait and an advanced age at disease onset (67 years) compared to the previously reported PNLA/PSP-PAGF cases rendered this possibility unlikely [19]. Moreover, as mentioned above, this case clearly fulfilled the diagnostic criteria of PSP - high density of neurofibrillary tangles (NFT) and neuropil threads (NT) in three out of four cardinal nuclei: substantia nigra, globus pallidus and pons and a moderate-to-high density of NFT and NT three out of four of secondary areas (striatum, oculomotor complex and dentate nucleus of cerebellum) -and did not resemble the distribution of pathological changes in PNLA/PSP-PAGF [2, 11, 19, 20].

### Clinical differential diagnosis

As hypokinesia in the left upper limb and hypomimia had initially been present, the diagnosis of PD had first been suggested. This diagnosis was questionable right from the beginning, since prominent PI was among the patient’s initial complaints, which does not develop until late into the disease in typical PD. Furthermore, tremor, present in many patients with PD and some patients with PSP-P was absent in this patient throughout the clinical course. The clear lack of response to levodopa therapy despite evidence for presynaptic dopaminergic degeneration by SPECT imaging suggested postsynaptic dopaminoceptive dysfunction in this patient and affirmed the doubt at the diagnosis of PD [9, 21, 22]. There was no reason to suggest multiple system atrophy (MSA) because ataxia and autonomic dysfunction were absent [9, 22]. A corticobasal syndrome (CBS) might have been considered by year 9, since apraxic features occurred in both hands. However, neither dystonia, myoclonus, nor alien limb phenomenon as additional cortical features were present, the motor signs were strikingly symmetric by then, and the average survival time of CBS patient (7.9 years) had passed by far at this time, rendering the diagnosis of CBS unlikely [9, 21, 23]. Brain imaging did not show vascular lesions of the basal ganglia and brainstem suggestive of vascular parkinsonism. Slowing of vertical saccades allowed the diagnosis of possible PSP according to the NINDS-SPSP criteria in the 9^th^ year of his illness. With development of vertical SNGP, the patient fulfilled the criteria for probable PSP in the 11^th^ year [5]. Cognitive changes typically observed in PSP, including apathy and reduced verbal fluency were not observed until 10 years after onset in this patient [21, 24]. Predominant mesencephalic atrophy clearly below the threshold suggestive of PSP [25] has been demonstrated by MRI in the 12^th^ year Since data from prior MRI is not available, it cannot be verified if structural imaging had allowed to suggest PSP at an earlier time point than the clinical features. As typical for PSP, the patient developed dysphagia in the terminal phase and died of aspiration pneumonia. Compared to the average disease duration of PSP (8.7 years) [10], the patient experienced an exceptionally long disease duration of 15 years, but individual cases with survival as long as 16 years have been previously reported [3].

In summary, this clinically well documented case strengthens the concept that individual patients with definite PSP can present with predominant progressive PI (PSP-PI predominance type), as it had been previously proposed on the basis of a retrospective case series. This observation further expands the clinical spectrum of definite PSP and underlines the need to adapt the clinical diagnostic criteria to allow diagnosing syndromes suggestive for PSP, such as PSP-PI, as early as possible.

## Abbreviations

CB: Coiled bodies
CBS: Corticobasal syndrome
FTD: Frontotemporal dysfunction
MRI: Magnetic resonance imaging
MSA: Multisystem atrophy
NFT: Neurofibrillary tangles
NT: Neuropil threads
PAGF: Pure akinesia with gait freezing
PD: Parkinson’s disease
PI: Postural instability
PNFA: Pallido-nigro-luysial atrophy
PSP: Progressive supranuclear palsy
PSP-PI: PSP with predominating postural instability
TA: Tufted astrocytes
